# Leadership training and quality improvement of interdisciplinary rounds in the ICU

**DOI:** 10.1186/cc12460

**Published:** 2013-03-19

**Authors:** EC Ten Have, JE Tulleken

**Affiliations:** 1 Universitair Medical Center Groningen, the Netherlands

## Introduction

The development of patient-centered care by interdisciplinary teams in the ICU has focused attention on leadership behavior. The purpose of this intervention study was to measure the effect of leadership training on the quality of performed interdisciplinary rounds (IDRs) in the ICU.

## Methods

In this nonrandomized intervention study, participants included nine intensive care medicine fellow trainees (intervention group) and 10 experienced intensivists (control group). Participants in the intervention and control groups previously were untrained in leading IDRs in the ICUs. After each participant led an IDR that was videotaped, the fellow trainees participated in a 1-day leadership training, which was consistent with principles of adult learning and behavioral modeling. After training, each fellow trainee led another IDR that was videotaped. Quality of the performed IDRs was measured by review of videotapes of the 19 IDRs lead by 19 intensivists, including 198 patient discussions subdivided into four ICUs, and assessment with the IDR-Assessment Scale.

## Results

Comparison of the intervention versus control groups shows that the intervention group has more yes scores on the IDR-Assessment Scale than the control group. This difference was significant in 12 of the, in total, 19 quality indicators.

## Conclusion

Quality of leadership will be reliably trained and measured in the context of IDRs in ICUs. Training in a simulation environment, with real-life IDR scenarios including conflicting situations, and workplace-based feedback in the preparation and feedback phases, appears to be effective to train leadership behaviour.
